# Colitis after checkpoint blockade: A retrospective cohort study of melanoma patients requiring admission for symptom control

**DOI:** 10.1002/cam4.2397

**Published:** 2019-07-09

**Authors:** Michael S. Hughes, Hui Zheng, Leyre Zubiri, Gabriel E. Molina, Steven T. Chen, Meghan J. Mooradian, Ian M. Allen, Kerry L. Reynolds, Michael Dougan

**Affiliations:** ^1^ Harvard Medical School Boston Massachusetts; ^2^ Department of Medicine Massachusetts General Hospital Boston Massachusetts; ^3^ Department of Dermatology Massachusetts General Hospital Boston Massachusetts; ^4^ Massachusetts General Hospital Cancer Center Boston Massachusetts; ^5^ Division of Gastroenterology Massachusetts General Hospital Boston Massachusetts

**Keywords:** adverse effects, checkpoint inhibition, colitis, CTLA‐4, hospitalization, immunotherapy, melanoma, programmed cell death 1

## Abstract

**Background:**

Immune checkpoint inhibitors (CPIs) have revolutionized oncologic therapy but can lead to immune‐related adverse events (irAEs). Corticosteroids are first‐line treatment with escalation to biologic immunosuppression in refractory cases. CPI‐related gastroenterocolitis (GEC) affects 20%‐50% of patients receiving CPIs and can carry significant morbidity and mortality. Severe CPI‐related GEC is not well‐described. We present the clinical characterization of all CPI‐related GEC requiring admission at a single institution.

**Methods:**

Clinical, laboratory, radiographic, and endoscopic data were extracted from charts of all melanoma patients ≥18 years of age admitted to one institution for CPI‐related GEC, from February 5, 2011 to December 13, 2016. Patients were followed until December 31, 2017 for further admissions. Survival, outcomes, and pharmaceutical‐use analyses were performed.

**Results:**

Median time‐to‐admission from initial CPI exposure was 73.5 days. Median length of stay was 4.5 days. About 50.0% required second‐line immunosuppression. Readmission for recrudescence occurred in 33.3%. Common Terminology Criteria for Adverse Events (CTCAE) grade was not significantly associated with outcomes. Hypoalbuminemia (*P* = 0.005), relative lymphopenia (*P* = 0.027), and decreased lactate dehydrogenase (*P* = 0.026) were associated with second‐line immunosuppression. There was no difference in progression‐free survival (PFS) or OS (*P* = 0.367, 0.400) for second‐line immunosuppression. Subgroup analysis showed that early corticosteroid administration (*P* = 0.045) was associated with decreased PFS.

**Conclusions:**

Severe CPI‐related GEC typically manifests within 3 months of immunotherapy exposure. Rates of second‐line immunosuppression and readmission for recrudescence were high. CTCAE grade did not capture the degree of severity in our cohort. Second‐line immunosuppression was not associated with poorer oncologic outcomes; however, early corticosteroid exposure was associated with decreased PFS. Further investigation is warranted.

## 1 INTRODUCTION

Immune checkpoint inhibitors (CPIs) have revolutionized cancer therapy over the past decade.^1^, ^2^ CPIs are clinically associated with durable responses in a wide range of cancers, including those of any origin with microsatellite instability or mismatch‐repair deficiencies, and the use of CPIs is rapidly growing.^3^, ^4^, ^5^, ^6^, ^7^ This novel class of agents enhances adaptive immune responses to cancer through inhibition of major T‐lymphocyte coinhibitory pathways that otherwise block “immune escape” mechanisms.^2^

Autoimmune‐like syndromes, termed immune‐related adverse events (irAEs), are becoming increasingly common as more patients are treated with CPIs.^1^, ^7^, ^8^, ^9^, ^10^, ^11^, ^12^ Though any organ system can be affected, irAEs typically involve the skin, gastrointestinal tract, and lungs.^13^ The severity and spectrum of irAEs are related to the specific pathway targeted, with inhibition of cytotoxic T‐lymphocyte antigen‐4 (CTLA‐4) generally having more frequent and more severe irAEs compared to inhibitors of programmed cell death‐1 (PD‐1) or its ligand (PD‐L1).^1^ Combination CPI therapy leads to more significant irAEs than either therapy alone.^12^

Gastroenterocolitis (GEC) is among the most common and severe irAEs associated with CPIs.^1^, ^12^, ^14^, ^15^ Depending on the targeted pathway, 20%‐50% of patients receiving commercially available CPI monotherapy develop some form of CPI‐related GEC, and 2%‐10% develop severe disease; rarely, bowel perforations can occur.^6^, ^8^, ^16^, ^17^, ^18^ Combination CPI therapy results in 46%‐51% of the exposed patients developing CPI‐related GEC, with 8%‐18% developing a severe form.^9^ CPI‐related GEC is accompanied by significant morbidity and substantial cost.^19^ First‐line treatment consists of immunosuppression with high‐dose corticosteroids (1‐2 mg/kg daily of prednisone or equivalent).^7^, ^20^ Tumor necrosis factor alpha (TNFα) inhibitors such as infliximab are standard second‐line treatment.^7^ Clinically, one of the most important factors complicating management of CPI‐related GEC is whether a patient responds to first‐line corticosteroids, and thus predictors of the need for second‐line immune suppression are of considerable clinical interest.

Severe CPI‐related GEC requiring hospitalization often necessitates acute intervention and forces oncologists to make difficult choices regarding further immunotherapy, including discontinuation of treatment. There are few published studies, however, describing a consolidated patient cohort hospitalized for the condition. We thus aimed to clinically characterize CPI‐related GEC requiring hospitalization in a retrospective cohort study, focusing on clinical factors that are associated with the requirement for secondary immune suppression.

## 2 METHODS

### 2.1 Ethics

This study was approved by the Partners Human Research Committee, the Institutional Review Board of the Massachusetts General Hospital (MGH).

### 2.2 Patients

**Figure 1:**
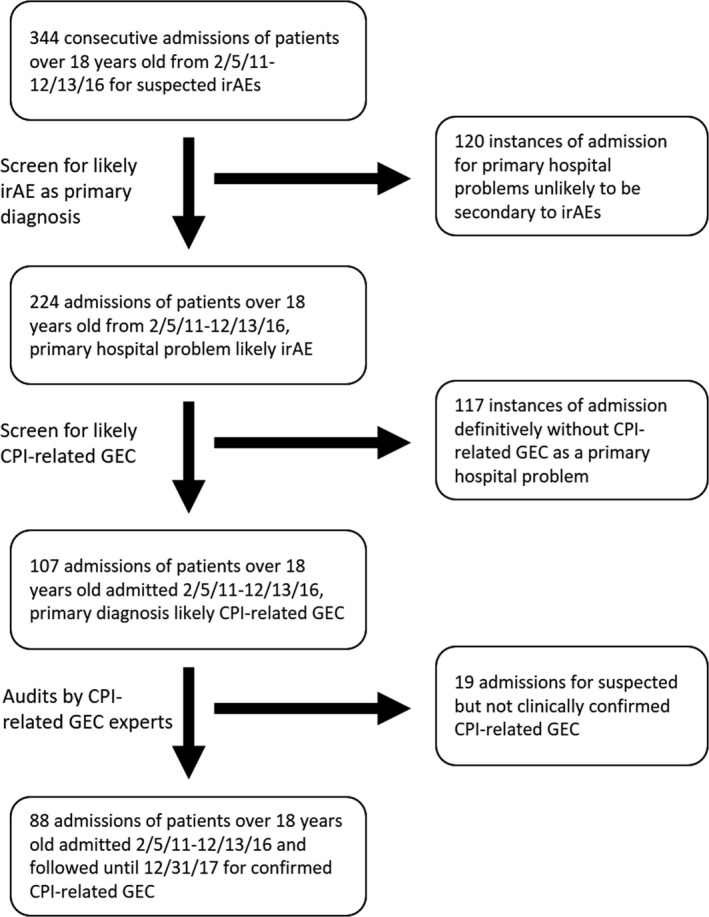
Process diagram depicting the generation of the patient cohort upon which descriptive and analytic statistics were performed. Patients were excluded at the initial screening for the following reasons: under 18 years of age; admitted for primary hospital problems unlikely to be secondary to irAEs. Patients were excluded at the second screening for the following reasons: complete absence of diarrhea, nausea/vomiting, or abdominal pain; otherwise admitted for primary hospital problems definitively not secondary to CPI‐related GEC. Patients were excluded at the audits stage by expert opinion after careful review of the electronic medical record. CPI, checkpoint inhibitor; GEC, gastroenterocolitis; irAEs, immune‐related adverse events

We identified all patients ≥18 years of age with stage III/IV melanoma hospitalized at Massachusetts General Hospital for expert‐confirmed CPI‐related GEC from February 05, 2011 to December 13, 2016; patients were followed for further admissions until December 31, 2017 (MGH Research Patient Data Registry). CPI‐related GEC was defined as clinical and/or histopathologic evidence of gastrointestinal inflammation best explained by prior CPI exposure. Diagnoses were confirmed by two reviewers with expertise in CPI complications (Figure 1). The cohort was divided into two comparison cohorts based on the use of corticosteroids alone, or combination with second‐line immune suppression for the treatment of CPI‐related GEC. The decision to use second‐line immune suppression was made by the treating medical team, and did not follow an explicit protocol; in general, the decision to start secondary immune suppression was made based on (a) lack of sufficient initial response to corticosteroids; and (b) recurrence of colitis symptoms upon corticosteroid taper. Treatment decisions were reviewed by two people with expertise in CPI complications and the use of secondary immune suppression was confirmed.

### 2.3 Data collection

We extracted clinical, laboratory, radiographic, and endoscopic data from electronic medical records (Table S1). The National Cancer Institute's Common Terminology Criteria for Adverse Events (CTCAE), version 4.0 was used for adverse event classification. Two independent database audits confirmed high accuracy.

### 2.4 Endpoints

Primary endpoints were progression‐free survival (PFS) and overall survival (OS). Secondary endpoints were length of stay (LOS), rate of readmission, and time to GEC resolution (grade 1 symptoms or better).

### 2.5 Statistical analysis

Patients were grouped in three primary ways for analysis: by CTCAE; by whether or not they received second‐line immunosuppression; and by whether they received corticosteroids earlier or later in their disease course. Descriptive statistics were displayed using Microsoft Excel 2016 (Microsoft Corporation, Redmond, Washington, DC). Statistical analysis was performed using SAS Studio (version 9.4M6, SAS Institute, Cary, NC). Data are expressed as “mean ± standard deviation,” “mean ± standard error,” or “median (range)” where appropriate. P‐values are two‐sided, with α = 0.05.

The chi‐squared test or Fisher's exact test and the ANOVA method or the Student's *t* test were employed where appropriate. Survival curves were generated using Kaplan‐Meier analysis. Log‐rank and Wilcoxon testing are reported where appropriate. Survival was measured from CPI exposure date to date of death, date of transition to hospice, or censored date. Date of death or transition to hospice was determined by electronic medical record review. Date of cancer progression was defined as the date imaging was performed showing progressive disease. Median follow‐up time was 28.0 months. Twenty‐eight patients transitioned to hospice care and/or died during the study interval. With a sample size of 30 in each subgroup, a follow‐up time of 60 months, and a median PFS of 10.5 months in those who did not receive second‐line immunosuppression and 30.5 months in those who did receive second‐line immunosuppression, we calculated that our study has over 80% power to detect a survival difference between populations using a two‐sided log‐rank test at a significance level of 0.05.

## 3 RESULTS

### 3.1 Characteristics and typical hospital course of severe CPI‐related GEC

Baseline characteristics are summarized in Table 1. Sixty patients with advanced melanoma, totaling 88 admissions, were hospitalized for CPI‐related GEC from June 1, 2011 to December 31, 2017. Average age on admission was 65 years; 38/60 (63%) patients were male and 52/60 (86.7%) had stage IV melanoma. Metastasis to the liver and the gastrointestinal tract were 35.0% (21/60, 35.0%) and 21.7% (13/60, 21.7%), respectively. Median Eastern Cooperative Oncology Group (ECOG) performance status was 0 at the time of CPI initiation.

**Table 1:** Characteristics of the patients at baseline

	Overall	No use of second‐line immunosuppression	Use of second line immunosuppression	*P*‐value
Number of patients	60	30/60 (50.0%)	30/60 (50.0%)	1.000
Number of admissions	88	35/88 (39.8%)	53/88 (60.3%)	0.288
Age in years (mean ± SD)	65.1 ± 12.2	67.9 ± 12.3	62.4 ± 11.6	0.080
Sex (M:F)	38:22	18:12	20:10	0.592
ICI regimen				
Ipilimumab	28/60 (46.7%)	9/30 (30.0%)	19/30 (63.3%)	0.010^a^
Pembrolizumab	7/60 (11.7%)	6/30 (20.0%)	1/30 (3.3%)	0.103
Nivolumab	1/60 (1.7%)	1/30 (3.3%)	0/30 (0.0%)	1.000
Combination	24/60 (40.0%)	14/30 (46.7%)	10/30 (33.3%)	0.292
Other	2/60 (3.3%)	1/30 (3.3%)	1/30 (3.3%)	1.000
Tumor stage				
II	1/60 (1.7%)	1/30 (3.3%)	0/30 (0.0%)	0.011^a^
III	7/60 (11.7%)	0/30 (0.0%)	7/30 (23.3%)	
IV	52/60 (86.7%)	29/30 (96.7%)	23/30 (76.7%)	
Prior therapies				
Median number of prior therapies (IQR)	2 (1‐3)	1 (1‐2)	2 (1‐3)	0.076
Resection	46/60 (76.7%)	22/30 (73.3%)	24/30 (80.0%)	0.542
Radiation	25/60 (41.7%)	11/30 (36.7%)	14/30 (46.7%)	0.432
Pegylated interferon	12/60 (20.0%)	4/30 (13.3%)	8/30 (26.7%)	0.197
Targeted inhibitor	15/60 (25.0%)	7/30 (23.3%)	8/30 (26.7%)	0.766
Chemotherapy	2/60 (3.3%)	2/30 (6.7%)	0/30 (0.0%)	0.492
CPI	8/60 (13.3%)	3/30 (10.0%)	5/30 (16.7%)	0.706
Gastrointestinal metastases				
Liver	21/60 (35.0%)	11/30 (36.7%)	10/30 (33.3%)	0.787
Other	13/60 (21.7%)	10/30 (33.3%)	3/30 (10.0%)	0.028^a^
Median ECOG performance status at initial CPI administration (IQR)	0 (0‐1)	0 (0‐1)	1 (0‐1)	0.358

Note

Selected typical characteristics of patients with CPI‐related GEC requiring admission. Univariate analysis displayed. The *P*‐value was calculated by ANOVA for numerical covariates and chi‐squared test or Fisher's exact for categorical covariates, where appropriate. The *P*‐value for survival analysis was determined with log‐rank testing.

Abbreviations: CPI, checkpoint inhibitor; CTCAE, common terminology criteria for adverse events; ECOG, Eastern cooperative oncology group; IQR, interquartile range; SD, standard deviation

a Statistically significant at α < 0.05

Twenty‐eight of sixty patients (47%) received ipilimumab monotherapy; 24/60 (40%) received combination CPI. Seven patients (12%) received pembrolizumab alone, one received nivolumab alone (2%), and two (3%) received alternative combinations. Median number of prior therapies was 2. Previous treatment with CPIs was uncommon (8/60, 13.3%) and no patients had had prior admissions for irAEs.

Admissions occurred a median of 73.5 days (range: 18.0‐1075.0) after first CPI dose. Presenting symptoms included diarrhea (83/88, 94%), nausea and/or vomiting (32/88, 36%), abdominal pain (37/88, 42%), melena/hematochezia (18/88, 20%), and fecal incontinence (5/88, 6%). In 49/88 admissions (55.7%), corticosteroids had been prescribed prior to admission. Admission chemistries and blood counts were typically within or near the normal range. Patients showed a mild lymphopenia (average 14.6%, 1120 cells/mL), mild anemia (average hemoglobin 12.8 g/dL), and hypoalbuminemia (average 3.6 g/dL). Erythrocyte sedimentation rate (ESR) (31.1 mm/h) and C‐reactive protein (CRP) (3.3 mg/L) were slightly elevated. One patient tested positive for *Clostridioides difficile* toxin, but the presentation was not consistent with isolated *Clostridioides difficile* colitis.

Cross‐sectional imaging was abnormal in 20/38 patients (52.6%). Diagnostic endoscopy was performed during 79/88 admissions (89.8%; 69 admissions with either an upper or lower endoscopy, 10 with both). Mucosal inflammation was found in 57/79 endoscopies (72.2%). Nearly all admitted patients received corticosteroids (57/60, 95.0%), with most instances of admission (77/88, 87.5%) involving at least 1 mg/kg prednisone or equivalent; 70/88 (79.5%) received intravenous high‐dose corticosteroid. Three of sixty (5.0%) experienced spontaneous symptom resolution without immunosuppression.

Half of our cohort (30/60, 50.0%) ultimately received second‐line immunosuppression after their treating team determined that corticosteroid treatment was insufficiently effective. In general, patients who received second‐line immunosuppression had experienced either persistent symptoms despite high‐dose corticosteroids or at least one episode of symptom recrudescence upon attempted corticosteroid taper. Most patients received infliximab (28/30, 93.3%). Emergent bowel resection occurred in two admissions (2.3%), and exploratory laparotomy in one (1.1%). Tables 1 and 2 display the differential associations between selected variables and second‐line immunosuppression. Ipilimumab monotherapy (*P* = 0.010), stage III disease postresection (*P* = 0.011), and the absence of gastrointestinal metastases (*P* = 0.028) were associated with second‐line immunosuppression. Patients who received second‐line immunosuppression had lower serum albumin (*P* = 0.005), lactate dehydrogenase (LDH) (*P* = 0.026), and relative lymphocyte counts (*P* = 0.027) (Table 2). They also tended to be younger with a higher median number of prior oncologic therapies and more weight loss (Table S2).

**Table 2:** Selected features of CPI‐related GEC presentation and initial diagnostic approach

	Overall	No use of second‐line immunosuppression	Use of second‐line immunosuppression	*P*‐value
Time to presentation, days				
Mean ± SD	133.1 ± 199.9	119.8 ± 138.8	141.8 ± 232.5	0.616
Median	73.5	93.0	70.0	
Presenting signs and symptoms				
Diarrhea	83/88 (94.3%)	33/35 (94.3%)	50/53 (94.3%)	1.000
Nausea and/or vomiting	32/88 (36.4%)	10/35 (28.6%)	22/53 (41.5%)	0.217
Abdominal pain	37/88 (42.1%)	13/35 (37.1%)	24/53 (45.3%)	0.449
Melena/hematochezia	18/88 (20.5%)	6/35 (17.1%)	12/53 (22.6%)	0.531
Fecal incontinence	5/88 (5.7%)	3/35 (8.6%)	2/53 (3.8%)	0.383
Other	32/88 (36.4%)	14/35 (40.0%)	18/53 (34.0%)	0.564
Percent weight change from baseline	−6% ± 7%	−5% ± 5%	−6% ± 8%	0.275
Median CTCAE symptom grade (IQR)	3 (3‐3)	3 (2‐3)	3 (3‐3)	0.198
Median ECOG Performance Status at admission (IQR)	1 (1‐2)	1 (1‐2)	1 (1‐2)	0.897
Laboratory results at admission: mean ± SD				
Routine chemistries	No significant abnormalities	No significant abnormalities	No significant abnormalities	>0.05
Albumin (g/dL)	3.6 ± 0.6	3.8 ± 0.6	3.5 ± 0.6	0.005^a^
Lactate dehydrogenase (U/L)	251.7 ± 28.4	309.5 ± 273.1	189.9 ± 69.7	0.026^a^
Erythrocyte sedimentation rate (ESR) (mm/h)	31.1 ± 28.4	31.4 ± 26.8	30.9 ± 31.2	0.976
C‐reactive protein (CRP) (mg/L)	3.3 ± 2.3	4.2 ± 2.7	2.9 ± 2.0	0.294
Complete Blood Count (CBC)	No significant abnormalities	No significant abnormalities	No significant abnormalities	>0.05
Lymphocytes, relative (%)	14.6 ± 2.1	17.2% ± 10.0%	12.9% ± 8.0%	0.027^a^
Lymphocytes, absolute (K cells/mL)	1.12 ± 0.71	1.31 ± 0.64	1.11 ± 0.74	0.193
Corticosteroid use at admission	49/88 (55.7%)	17/35 (48.6%)	32/53 (60.4%)	0.275
Diagnostic studies on admission				
Radiographic signs of gastrointestinal inflammation	20/38 (52.6%)	3/10 (30.0%)	17/28 (60.7%)	0.144
Endoscopic signs of gastrointestinal inflammation	54/69 (78.2%)	23/31 (74.2%)	31/38 (81.6%)	0.459

Note

Selected presenting features of CPI‐related GEC requiring hospitalization, together with components and results of initial diagnostic approach. Inadequate bowel preparations obscuring visual examination occurred at a negligible rate. Univariate analysis displayed. The *P*‐value was calculated by ANOVA for numerical covariates and chi‐squared test or Fisher's exact for categorical covariates, where appropriate. Routine chemistries include the following: serum sodium (mmol/L), serum potassium (mmol/L), serum chloride (mmol/L), blood urea nitrogen (mg/dL), serum creatinine (mg/dL), lactate (mmol/L). Complete blood count includes the following: white blood cells (K cells/mL), hematocrit (%), hemoglobin (g/dL), platelets (K cells/mL). Other symptoms included fatigue, night sweats, abdominal bloating, chills, dysphagia, and hypotension. Endoscopy includes: esophagogastroduodenoscopy, flexible sigmoidoscopy, and colonoscopy.

Abbreviations: CPI, checkpoint inhibitor; CTCAE, common terminology criteria for adverse events; ECOG, Eastern cooperative oncology group; IQR, interquartile range; SD, standard deviation.

a Statistically significant at α < 0.05.

The need for second‐line immunosuppression was not associated with CTCAE grade, type of CPI treatment, ECOG performance status, corticosteroid use prior to admission, or the presence/absence of radiographic or endoscopic abnormalities (Table 2). The presence of melena or hematochezia on admission was associated with CTCAE grade ≥2 (Table 3).

**Table 3:** Selected admission‐specific variables by CTCAE grade

	CTCAE 1	CTCAE 2	CTCAE 3	CTCAE 4	*P*‐value
Number of cases	1	13	70	3	0.253
Clinical features					
Diarrhea	1/1 (100.0%)	12/13 (92.3%)	67/70 (95.7%)	2/3 (66.7%)	0.160
Nausea/vomiting	0/1 (0.0%)	5/13 (38.5%)	26/70 (37.1%)	1/3 (33.3%)	1.000
Abdominal pain	1/1 (100.0%)	5/13 (38.5%)	30/70 (42.9%)	2/3 (66.7%)	0.889
Melena/hematochezia	1/1 (100.0%)	0/13 (0.0%)	15/70 (21.4%)	2/3 (66.7%)	0.042^a^
Fecal incontinence	1/1 (100.0%)	0/13 (0.0%)	5/70 (7.1%)	0/3 (0.0%)	1.000
Other	0/1 (100.0%)	7/13 (53.9%)	24/70 (34.3%)	1/3 (33.3%)	0.525
Endoscopy abnormalities	N/A	7/10 (70.0%)	44/56 (78.6%)	2/2 (100.0%)	0.808
Time to admission in days					
Mean ± SD	59 ± N/A	133.4 ± 198.2	137.8 ± 207.7	85 ± 16.8	0.954
Median	59	74	76.5	91	
Length of stay in days per admission					
Mean ± SD	6 ± N/A	5.5 ± 4.3	5.5 ± 4.0	12.7 ± 2.9	0.033^a^
Median	6	4	4	11	
GEC symptom return to grade 1 or baseline after first admission					
At 1 mo postdischarge	1/1 (100.0%)	6/7 (85.7%)	42/46 (91.3%)	0/1 (0.0%)	0.098
At 3 mo postdischarge	0/0 (N/A)	5/5 (100.0%)	40/41 (97.6%)	2/2 (100.0%)	1.000

Note

Selected variables regarding patients with CPI‐related GEC, stratified by CTCAE grade upon presentation. Univariate analysis displayed. The *P*‐value was calculated by ANOVA for numerical covariates and chi‐squared test or Fisher's exact for categorical covariates, where appropriate.

Abbreviations: CPI, checkpoint inhibitor; CTCAE, Common Terminology Criteria for Adverse Events; GEC, gastroenterocolitis; NE, not estimable; SD, standard deviation.

a Statistically significant at α < 0.05.

### 3.2 Endpoints

#### 3.2.1 Primary endpoint assessment

**Figure 2:**
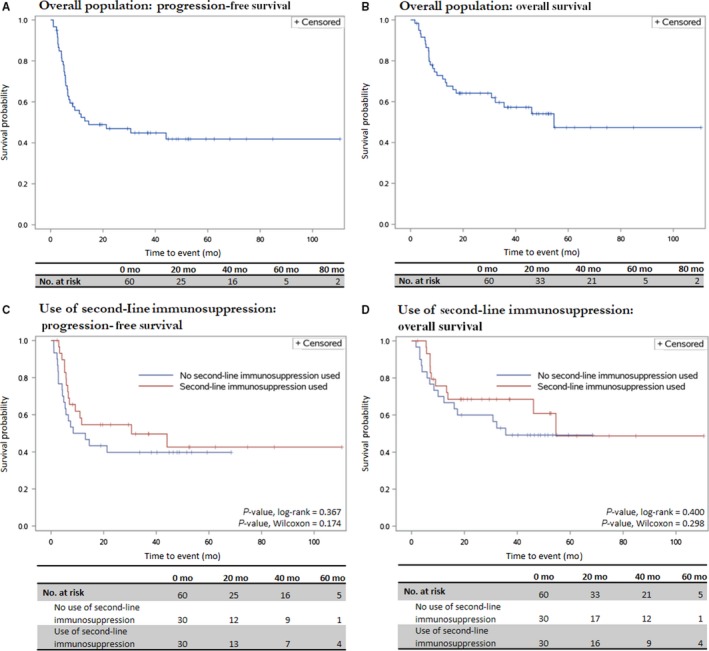
Kaplan‐Meier survival graphs depicting oncologic outcomes. A, PFS, overall population. B, OS, overall population. C, PFS, stratified by use of second‐line immunosuppression. D, OS, stratified by use of second‐line immunosuppression. CI, confidence interval; OS, overall survival; PFS, progression‐free survival

We characterized oncologic outcomes and associations with second‐line immunosuppression (Table 4, Figure 2). Overall mean PFS and OS were 23.8 and 36.1 months, respectively (medians 14.5 and 54.6 months). Mean PFS and OS for patients without second‐line immunosuppression were 12.2 and 24.2 months (medians 10.8 and 35.6 months). Mean PFS and OS for those who received second‐line immunosuppression were 26.4 and 39.4 months (medians 30.6 and 54.6 months). No significant differences in second‐line immunosuppression were observed in PFS (*P* = 0.367 log‐rank, 0.174 Wilcoxon) or OS (*P* = 0.400 log‐rank, 0.298 Wilcoxon).

**Table 4:** Characteristics of hospital and postdischarge course

	Overall	No use of second‐line immunosuppression	Use of second‐line immunosuppression	*P*‐value
Length of stay in days per admission				
Mean ± SD	5.8 ± 4.2	5.2 ± 3.7	6.3 ± 4.5	0.226
Median	4.5	4.0	5.0	
Readmissions for recrudescence				
Number of patients requiring >1 readmission	20/60 (33.3%)	5/30 (16.7%)	15/30 (50.0%)	0.091
Number of patients requiring ≥1 readmission	6/60 (10.0%)	0/30 (0.0%)	6/30 (20.0%)	0.055
GEC symptom return to grade 1 or baseline after first admission				
At 1 mo postdischarge	49/56 (87.5%)	24/28 (85.7%)	25/28 (89.3%)	0.669
At 3 mo postdischarge	50/51 (98.0%)	22/23 (95.7%)	28/28 (100.0%)	0.451
Progression‐free Survival				
Mean ± SE	23.8 ± 2.5	12.2 ± 1.6	26.4 ± 3.6	0.367
Median (CI)	14.5 (6.6‐NE)	10.8 (4.8‐NE)	30.6 (6.5‐NE)	
Overall survival				
Mean ± SE	36.1 ± 2.9	24.2 ± 2.6	39.4 ± 4.2	0.400
Median (CI)	54.6 (30.8‐NE)	35.6 (12.2‐NE)	54.6 (13.7‐NE)	

Note

Characteristics of later hospital course and postdischarge course as primary and secondary endpoints of the study. GEC symptoms were inquired after at standard oncologic follow‐up visits. Of note, the total number of patients decreased over time, yielding decreasing denominators in “GEC symptom resolution after first admission.” Univariate analysis displayed. The *P*‐value was calculated by ANOVA for numerical covariates and chi‐squared test or Fisher's exact for categorical covariates, where appropriate.

Abbreviations: GEC, gastroenterocolitis; NE, not estimable; SD, standard deviation; SE, standard error.

**Figure 3:**
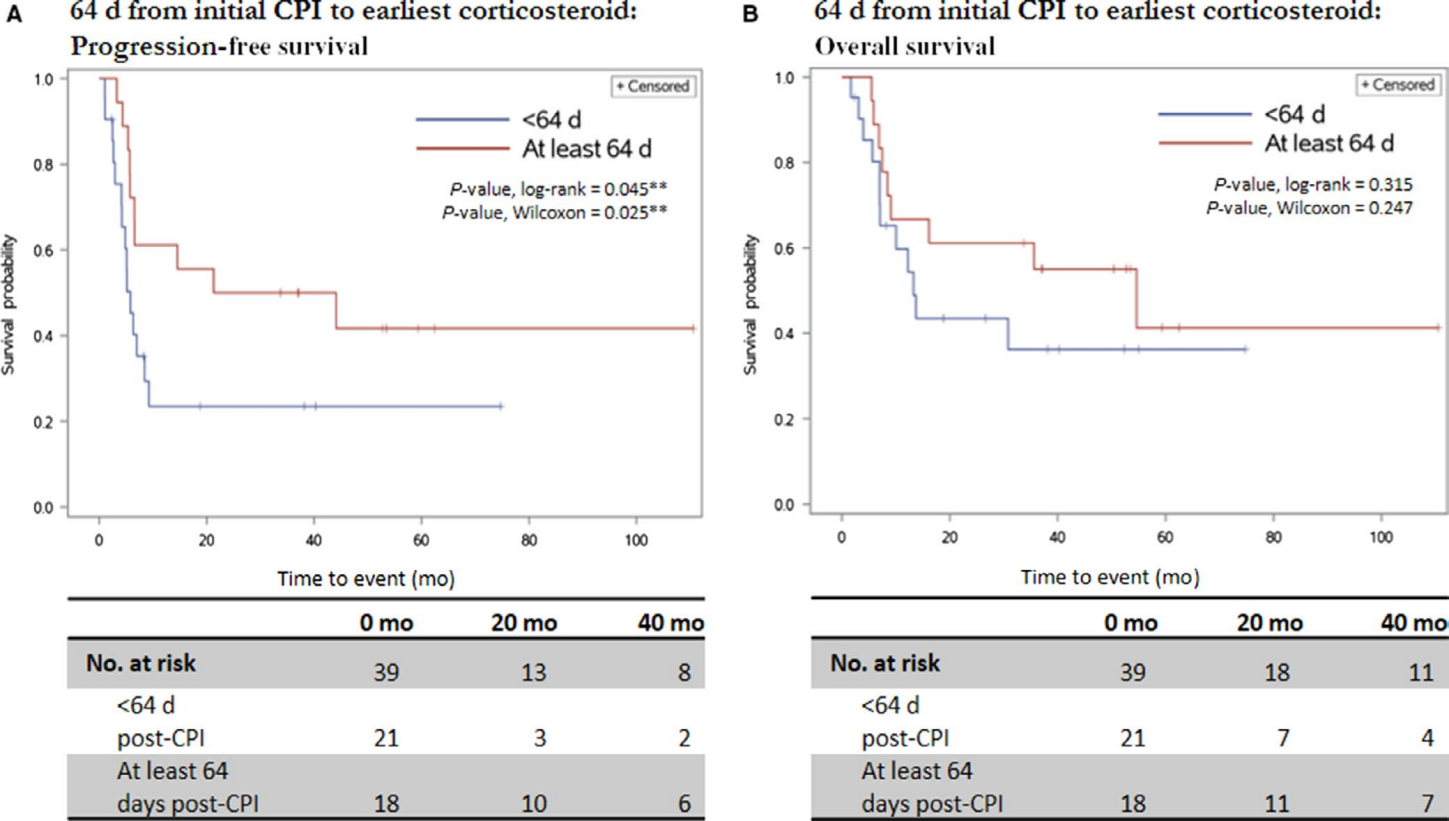
Kaplan‐Meier survival curves for oncologic outcomes in patients with stage IV melanoma who received at least two cycles of ipilimumab, stratified by GEC/corticosteroid exposure. Time threshold: 64 days. One patient had unclear corticosteroid dosing timing and was therefore not included in this analysis. **Denotes significance at α < 0.05. A, PFS, stratified by time from initial ICI administration to earliest GEC/corticosteroid exposure at any dose. B, OS, stratified by time from initial ICI administration to earliest GEC/corticosteroid exposure at any dose. GEC, gastroenterocolitis; OS, overall survival; PFS, progression‐free survival

We also examined oncologic outcomes with respect to the timing of GEC and subsequent corticosteroid exposure in patients with stage IV melanoma who received at least two cycles of ipilimumab, divided at median time to corticosteroid administration: “early exposure,” defined as receiving corticosteroids for GEC within 64 days after CPI administration; and “late exposure,” defined as receiving corticosteroids for GEC at least 64 days after CPI administration. Decreased PFS was significantly associated with early exposure (*P* = 0.045 log‐rank, 0.025 Wilcoxon) with no statistically significant effect on OS (Figure 3B). Cox proportional hazard modeling of the effect of corticosteroid administration timing on PFS, controlling for age at admission and patient sex, showed a hazard ratio of 2.26 (95% confidence interval: 1.00‐5.11, *P* = 0.051; Table S3). Similar analyses for other types of corticosteroid exposures revealed significant differences (Figure S1). The same analyses for the time interval between symptom onset and corticosteroid exposures revealed no significant differences in PFS or OS. By the same token, “early” admission was associated with poorer PFS at borderline significance (*P* = 0.133 log‐rank, 0.046 Wilcoxon) but showed no significant difference in OS. Timings of analyzed corticosteroid exposures were significantly collinear with time to admission (Figure S2).

#### 3.2.2 Secondary endpoint assessment

**Figure 4:**
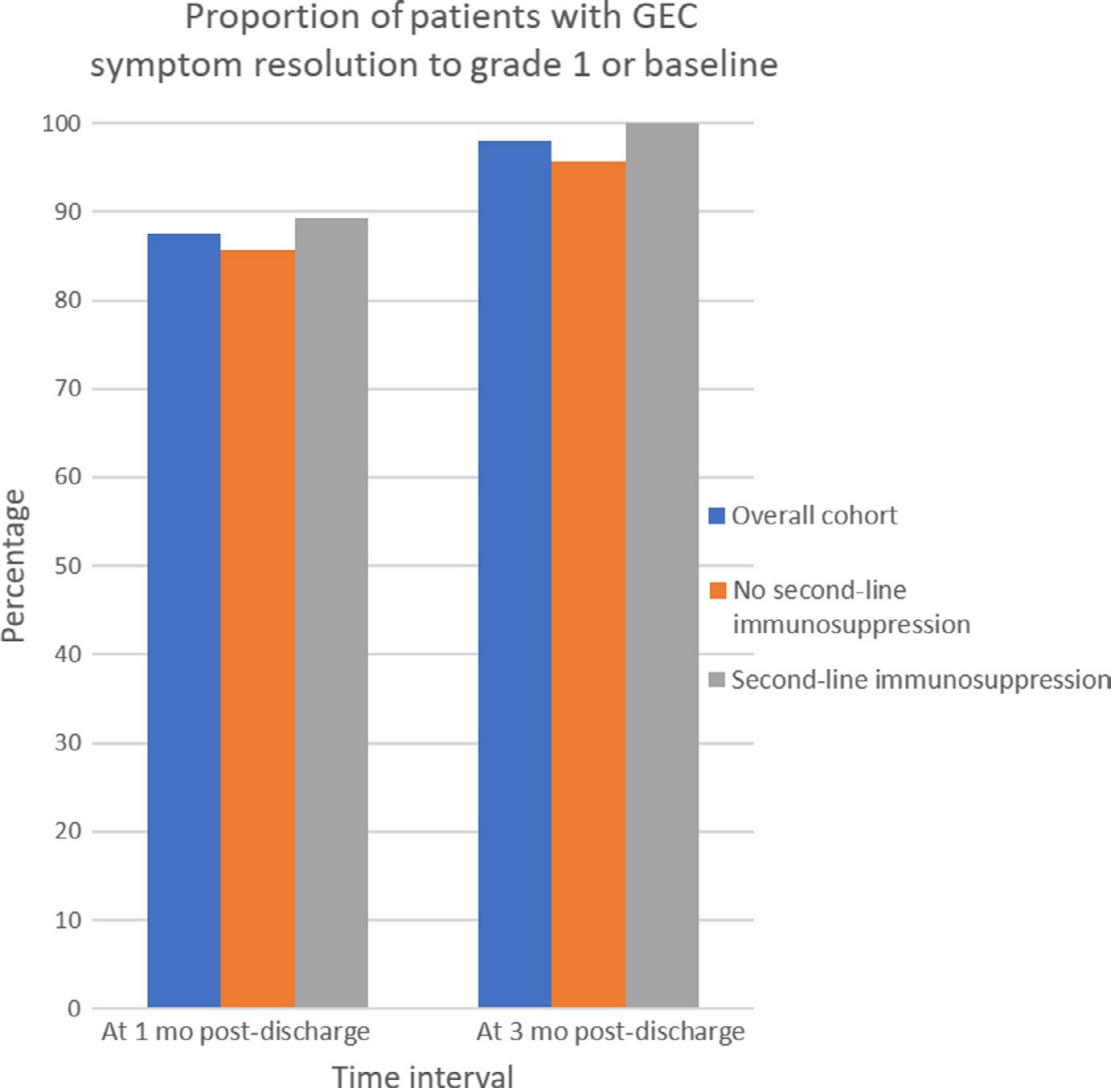
Proportions of patients with GEC symptom resolution to grade 1 or baseline, stratified by secondline immunosuppression. *P* > 0.05 by ANOVA. GEC, gastroenterocolitis

Average LOS was 5.8 ± 4.2 days; median LOS was 4.5 days. Readmission for GEC recrudescence was 33.3% (20/60); 30.0% of the cohort (18/60) were readmitted within 30 days. About 10.0% (6/60) required multiple readmissions. Maximum number of readmissions was three. GEC resolution rate was 87.5% (49/56) at 1 month postdischarge and 98.0% (50/51) at 3 months postdischarge (Figure 4); the same pattern was observed regardless of second‐line immunosuppression use. No differences in LOS or rate of readmission for GEC recrudescence between patients who received second‐line immunosuppression and those who did not were observed. CTCAE grade was overall not significantly associated with short‐term outcomes, but grade 4 severity was associated with LOS approximately 1 week longer than that of other grades (12.7 days vs 5.5 days, *P* = 0.033) and grade 2 cases tended to worsen within 1 month of discharge (Table 3). The small number of grade 4 cases precluded meaningful long‐term survival analysis. Patients who received second‐line immunosuppression tended to be readmitted more often (*P* = 0.091) and to require multiple readmissions (*P* = 0.055).

Early IV steroid exposure was associated with lower likelihood of readmission for recrudescence (*P* = 0.019). Any early steroid exposure showed lower likelihood of readmission for recrudescence but did not reach significance (*P* = 0.060). No differences were observed in LOS.

### 3.3 Additional analysis

Melanoma and irAE treatment strategies changed over our study period. We accordingly characterized patterns of second‐line immunosuppression use and time from admission to infliximab administration over 2011‐2017 in patients receiving ipilimumab‐containing regimens. Infliximab use did not vary significantly over the 6 years studied (Table 5). Likewise, no significant variation was noted in time to infliximab administration (Table 6). Mean time from initial admission to infliximab exposure intervals ranged from 11.3 days in January 2016 to December 2017 to 51.2 days in January 2014 to December 2015; median interval range was small, between 19 days in January 2016 to December 2017 and 20.5 days prior to January 2014 (*P* = 0.188).

**Table 5:** Univariate Analysis of Ipilimumab Administration by Use of Second‐Line Immunosuppression over Study Timespan

	Use of second‐line immunosuppression (%)	*P*‐value
Number of patients with anti‐CTLA‐4 containing regimen, 2011‐2017	29/52 (55.8%)	0.694
Prior to January 2013	13/18 (72.2%)	0.564
January 2014 to December 2015	7/23 (30.4%)	
January 2016 to December 2017	9/11 (81.8%)	

Note

Univariate analysis of change in selected variables over study's timespan. Analysis of ipilimumab use over time. Statistically significant differences not observed.

The *P*‐value was calculated by ANOVA for numerical covariates and chi‐squared test or Fisher's exact for categorical covariates, where appropriate.

CTLA‐4, cytotoxic T‐lymphocyte antigen‐4

Statistically significant at α < 0.05.

**Table 6:** Univariate analysis of time from first admission to TNFαi administration over study timespan

	Time to TNFαi (days)	*P*‐value
Prior to January 2014		0.188
Mean ± SD	18.1 ± 12.2	
Median	20.5	
January 2014 to December 2015		
Mean ± SD	51.2 ± 68.5	
Median	19.5	
January 2016 to December 2017		
Mean ± SD	11.3 ± 46.9	
Median	19	

Note

Univariate analysis of change in selected variables over study's timespan. Analysis of change in time interval from first admission to TNFαi administration, as surrogate for need for second‐line immunosuppression, over study's timespan. No statistically significant differences seen, including in results by individual year (not shown). The *P*‐value was calculated by ANOVA for numerical covariates and chi‐squared test or Fisher's exact for categorical covariates, where appropriate.

Abbreviations: SD, standard deviation; TNFαi: tumor necrosis factor alpha inhibitor.

Statistically significant at α < 0.05.

## 4 DISCUSSION

In this retrospective, single‐center study, we describe the typical disease course of CPI‐related GEC requiring hospitalization in patients with advanced melanoma, including postdischarge outcomes, and we identify factors associated with second‐line immunosuppression use for symptom control. Survival analysis suggests that while second‐line immunosuppression is not associated with worse PFS or OS, increased time from initial CPI to corticosteroid administration is significantly associated with increased PFS and is collinear with time to admission.

CPI‐related GEC is a common irAE accompanied by significant morbidity and mortality. One‐quarter of patients receiving anti‐CTLA‐4 therapy develop low‐grade GEC, while 11% experience severe GEC; 10%‐20% of patients exposed to anti‐PD‐1/PD‐L1 develop a low‐grade GEC, while 2% develop high‐grade symptoms.^1^, ^8^, ^9^, ^10^, ^13^, ^17^ As expected, combination therapy seems to have at least an additive, if not synergistic, effect on toxicity.^9^, ^10^ Empiric evidence in CPI‐related GEC suggests that gastrointestinal immune homeostasis is often significantly disrupted in the setting of CPI therapy,^1^ but pathogenesis is not fully understood. In general, the self‐targeting seen in irAEs is felt to be the result of an underlying predisposition to autoimmunity, with or without shared tumor‐tissue antigenicity, triggered by disinhibited T‐lymphocytes.^12^, ^21^ There seem to be multiple mechanisms through which such cells act to produce symptoms of autoimmune disease: for example, direct tissue infiltration is seen in vitiligo and myocarditis^21^, ^22^; autoantibody generation is seen in myasthenia gravis and meningoencephalitis^23^, ^24^; and toxicities mediated by inflammatory cytokines are seen in PD‐1 inhibitor‐induced cytokine release syndrome.^25^

From previously published data, CPI‐related GEC was typically present with diarrhea, abdominal pain, and evidence of gastrointestinal tract ulceration, similar to inflammatory bowel disease.^1^, ^12^ Symptom onset ranges from 6 weeks to several months after initial CPI administration. Inflammation most often affects the colon and can be concomitantly seen in stomach or small intestine; however, isolated gastritis or enteritis can also occur.^26^, ^27^ Diagnostic approaches include radiography and endoscopy with further histopathologic examination. Upon endoscopy, enteritis and gastritis are typically characterized by significant ulceration.^1^, ^12^, ^15^ There are two primary patterns of colitis reported: similar significant ulcerations or normal mucosa with microscopic damage.^1^ Management of CPI‐related GEC relies on high‐dose corticosteroids with escalation to infliximab, though alternative second‐line therapies are emerging.^7^, ^28^ The particular steroid administered does not seem to impact clinical course.^7^, ^29^ The prognostic implications of developing CPI‐related GEC on oncologic outcomes are uncertain; other irAEs have been associated with improved survival, but few data link GEC and antitumor effect.^30^, ^31^

From our analysis, we conclude that patients with severe CPI‐related GEC generally develop symptoms approximately 9 weeks post‐initial CPI, 1 week more than the median time to presentation for any‐severe CPI‐related GEC reported previously,^20^ and are admitted at a median of 10 weeks from initial CPI administration. Common clinical features include diarrhea, abdominal pain, and nausea and vomiting. A smaller proportion also manifested with melena or hematochezia. Compared with the reported presentation of nonsevere CPI‐related GEC, the severe form of the condition is associated with a higher incidence of melena and hematochezia.^15^, ^32^ Intriguingly, we report a lower prevalence of abdominal pain.^15^, ^32^

Diagnostic workup in the overall cohort was notable for the difference between radiography and endoscopy in revealing inflammation. The substantially lower rate at which imaging was performed may reflect adaptation of clinical practice to the fact that radiography was often unremarkable, whereas endoscopy was abnormal in most of the cases in which it was performed. Several recent investigations have suggested serologic markers for severe CPI‐related GEC development.^33^, ^34^, ^35^ In particular, high‐risk endoscopic findings including significant ulceration have been associated with the need for second‐line immunosuppression.^36^ In our cohort, second‐line immunosuppression was administered at a 50.0% frequency, a rate higher than the 22.5% rate in previous reports, likely reflecting the overall higher acuity of this cohort as admission to a tertiary care hospital was a requirement for study inclusion.^32^ Second‐line immunosuppression was initiated in consultation with an expert in GI‐related irAEs. The decision to pursue such a consultation occurred at the primary oncologist's discretion. All patients who received second‐line immunosuppression exhibited at least 24 hours of persistent severe GEC despite high‐dose corticosteroids or had demonstrated significant symptom recrudescence, though no specific criteria were used to make this treatment decision in our cohort. Our rate of surgical therapeutic intervention (3.4%) was comparable to that of prior investigations (6%), underscoring the importance of careful monitoring of patients with severe CPI‐related GEC.^32^

Regardless of second‐line immunosuppression, however, patients with severe CPI‐related GEC are high short‐term utilizers of healthcare services. The median LOS for severe CPI‐related GEC was comparable to that of the general cancer patient admission at a comparable institution.^37^ Rate of readmission for recrudescence was 33.3%, which is lower than reported overall rates of cancer patient readmission (43%).^37^ Our 30‐day rate of readmission for recrudescence (30.0%), however, was substantially higher than reported values for 30‐day unplanned readmission rates in cancer patients (14.9%).^38^ Nevertheless, if patients were not readmitted within the first 3 months postdischarge, symptoms almost invariably resolved (98.0%) during the same timeframe. Additionally, early IV steroids resulted in a lower readmission rate. Average PFS was comparable to published estimates for standard CPI regimens in advanced melanoma.^9^, ^39^

In our study, more severe relative lymphopenia, lower serum albumin, and lower LDH are significantly correlated with higher chance of second‐line immunosuppression use. The absolute differences are small; however, they may indicate the underlying mechanisms of disease. Hypoalbuminemia may result from enteric ulcerations that are associated with more severe GEC.^34^, ^35^, ^36^ The difference in relative lymphopenia we observed, without significant difference in absolute lymphopenia, may indicate increased neutrophilic production, driven perhaps in part by elevated interleukin‐17 levels^40^ but more likely by multiple cytokines generated in response to gastrointestinal mucosal compromise and subsequent immune activation by microbial products. Lower LDH may suggest that CPI‐related GEC which requires hospitalization and second‐line immunosuppression is actually a positive prognostic factor for oncologic response.^41^ The significant differences in second‐line immunosuppression for age, tumor stage, and the absence of non‐hepatic gastrointestinal tract metastases are most likely due to a heterogeneous population and dose‐dependent ipilimumab toxicity: during 2011‐2017, a small number of patients with high‐risk nonmetastatic melanoma were treated with adjuvant ipilimumab at an increased dose. High‐dose ipilimumab used as adjuvant therapy has been shown in stage III melanoma to prolong survival but also to increase the risk of irAE.^42^

We detected no significant difference in second‐line immunosuppression use or rate of readmission for CTCAE grade. Such findings demonstrate that substantial heterogeneity in severe CPI‐related GEC is not adequately captured by CTCAE alone: a more nuanced classification system with stronger correlation to second‐line immunosuppression use and readmission is needed. CTCAE grade 4 cases do present more frequently with melena or hematochezia, as might be expected if severity of mucosal ulceration is correlated with severity of disease. CTCAE grade 4 cases also stay approximately 1 week more in the hospital; however, this is most likely due to a distribution of CTCAE grades skewed toward 3.

Our survival analyses indicate that second‐line immunosuppression in severe CPI‐related GEC does not negatively impact oncologic outcomes and does not affect LOS or readmission frequency. Intriguingly, our findings suggest that, in a subset of patients, decreased time from CPI administration to GEC development and subsequent corticosteroids at any dose is linked to poorer PFS. In the same group of patients, decreased time from CPI administration to admission tended toward poorer outcomes as well but did not reach significance by log‐rank testing. The two variables were also shown to be highly colinear. Significant differences were noted at the 64‐day threshold; other exposure parameters showed borderline significance (Figure S1).

Patients who were admitted later for CPI‐related GEC and who thus received corticosteroids later may have benefited from receiving more immunotherapy, potentially accounting for the observed difference in PFS. However, the association between early corticosteroid administration and decreased PFS is also compatible with a model of cancer immunotherapy‐related autoimmune disease in which corticosteroids impact the antitumor response. Several prior retrospective studies have not found that steroids reduce antitumor immune activity.^43^ On the other hand, prior in vitro and animal experiments have shown that glucocorticoids block antibody‐dependent tumor cell destruction^44^; may abrogate interleukin‐1α‐mediated antitumor activity^45^; and upregulate CTLA‐4 in animal models of intracranial gliomas responsive to CTLA‐4 inhibition.^46^ A recent study concluded in addition that infliximab, in contrast to corticosteroids, had little to no negative impact on tumor‐infiltrating lymphocyte function.^47^

Whether or not such effects induced by corticosteroids are clinically important and remain unclear; however, a significant association between corticosteroid administration prior to immunotherapy initiation and poorer oncologic outcomes in patients with nonsmall‐cell lung cancer has been reported in a retrospective cohort study,^48^ and a recent retrospective study of ipilimumab‐induced hypophysitis in patients with advanced melanoma suggested that higher corticosteroid doses resulted in reduced OS.^49^ Our findings additionally suggest that corticosteroid timing may play a role in immunotherapy response, but further prospective study will be required to determine whether corticosteroids have a direct impact on antitumor responses. While early corticosteroid exposure may, to some extent, limit antitumor responses, high‐dose corticosteroids in acute irAEs nevertheless clearly constitute lifesaving first‐line treatment for many patients.^7^

As these were univariate survival analyses performed in a subset of patients, our findings may be confounded by multiple factors, including codependence. Our cohort's heterogeneous CPI exposure is another potential confounding variable. Other limitations include a retrospective study design, which does not allow for causality inference and is inherently confounded by survival bias. Small sample size limited our power to make statistical observations. Our 6‐year study period encompassed substantial changes in standard‐of‐care therapy for melanoma.^50^, ^51^ Of note, though many patients received ipilimumab monotherapy and few patients received PD‐1‐targeted monotherapies, combination CPI regimens are well‐represented, allowing for limited generalizability and indicating a need for further study.

## 5 CONCLUSIONS

Typical CPI‐related GEC requiring hospitalization manifests within 3 months of initial CPI with a distinct constellation of clinical features. Diagnostic workup shows multiple abnormalities, several of which may be associated with second‐line immunosuppression use. Overall, 50% of patients receive second‐line immunosuppression. Readmission for recrudescence is frequent. If patients are not readmitted for symptom recrudescence, however, their GEC will most likely substantially improve within 3 months postdischarge. Second‐line immunosuppression has no detrimental effect on oncologic outcomes, but corticosteroid timing may.

Further studies in CPI‐related GEC requiring hospitalization are needed to: (a) add nuance to the “classic” clinical description above; (b) construct a more granular grading system that accounts for inpatient disease severity; (c) corroborate our survival analysis findings; and (d) prospectively parse the survival impact of time from initial CPI exposure to corticosteroid administration apart from that of time on immunotherapy.

## CONFLICTS OF INTEREST

MD receives research funding from Novartis Pharmaceuticals, and is a consultant for Genentech. All remaining authors report no conflicts of interest.

## AUTHOR CONTRIBUTIONS

MSH, KLR, and MD were involved in all aspects of this study including database design, data collection, data analysis, interpretation, and writing the manuscript. HZ was involved in data analysis and editing the manuscript. LZ was involved in data collection and editing the manuscript. GEM, STC, IMA, and MJM were involved in editing the manuscript. KLR and MD oversaw all work performed.

## References

[1] Dougan M . Checkpoint blockade toxicity and immune homeostasis in the gastrointestinal tract. Front Immunol. 2017;8:1547. 29230210 10.3389/fimmu.2017.01547 PMC5715331

[2] Baumeister SH , Freeman GJ , Dranoff G , Sharpe AH . Coinhibitory pathways in immunotherapy for cancer. Annu Rev Immunol. 2016;34:539‐573. 26927206 10.1146/annurev-immunol-032414-112049

[3] Le DT , Uram JN , Wang HB , et al. PD-1 blockade in tumors with mismatch-repair deficiency. N Engl J Med. 2015;372(26):2509–2520. 26028255 10.1056/NEJMoa1500596 PMC4481136

[4] Koster BD , de Gruijl TD , van den Eertwegh AJ . Recent developments and future challenges in immune checkpoint inhibitory cancer treatment. Curr Opin Oncol. 2015;27(6):482‐488. 26352539 10.1097/CCO.0000000000000221

[5] Collin M . Immune checkpoint inhibitors: a patent review (2010–2015). Expert Opin Ther Pat. 2016;26(5):555‐564. 27054314 10.1080/13543776.2016.1176150

[6] Michot JM , Bigenwald C , Champiat S , et al. Immune‐related adverse events with immune checkpoint blockade: a comprehensive review. Eur J Cancer. 2016;54:139‐148. 26765102 10.1016/j.ejca.2015.11.016

[7] Brahmer JR , Lacchetti C , Schneider BJ , et al. Management of immune‐related adverse events in patients treated with immune checkpoint inhibitor therapy: American Society of Clinical Oncology Clinical Practice Guideline. J Clin Oncol. 2018;36(17):1714‐1768. 29442540 10.1200/JCO.2017.77.6385 PMC6481621

[8] Topalian SL , Sznol M , McDermott DF , et al. Survival, durable tumor remission, and long‐term safety in patients with advanced melanoma receiving nivolumab. J Clin Oncol. 2014;32(10):1020‐1030. 24590637 10.1200/JCO.2013.53.0105 PMC4811023

[9] Larkin J , Chiarion‐Sileni V , Gonzalez R , et al. Combined nivolumab and ipilimumab or monotherapy in untreated melanoma. N Engl J Med. 2015;373(1):23‐34. 26027431 10.1056/NEJMoa1504030 PMC5698905

[10] Hodi FS , Chesney J , Pavlick AC , et al. Combined nivolumab and ipilimumab versus ipilimumab alone in patients with advanced melanoma: 2‐year overall survival outcomes in a multicentre, randomised, controlled, phase 2 trial. Lancet Oncol. 2016;17(11):1558‐1568. 27622997 10.1016/S1470-2045(16)30366-7 PMC5630525

[11] Sznol M , Ferrucci PF , Hogg D , et al. Pooled analysis safety profile of nivolumab and ipilimumab combination therapy in patients with advanced melanoma. J Clin Oncol. 2017;35(34):3815‐3822. 28915085 10.1200/JCO.2016.72.1167

[12] Dougan M , Dranoff G , Cancer D . Immunotherapy: beyond checkpoint blockade. Ann Rev Cancer Biology. 2019;3(1):55–75. 10.1146/annurev-cancerbio-030518-055552 PMC10400018 37539076

[13] Pauken KE , Dougan M , Rose NR , Lichtman AH , Sharpe AH . Adverse events following cancer immunotherapy: obstacles and opportunities. Trends Immunol. 2019;40(6):511–523. 31053497 10.1016/j.it.2019.04.002 PMC6527345

[14] Beck KE , Blansfield JA , Tran KQ , et al. Enterocolitis in patients with cancer after antibody blockade of cytotoxic T‐lymphocyte‐associated antigen 4. J Clin Oncol. 2006;24(15):2283‐2289. 16710025 10.1200/JCO.2005.04.5716 PMC2140223

[15] Collins M , Michot JM , Danlos FX , et al. Inflammatory gastrointestinal diseases associated with PD‐1 blockade antibodies. Ann Oncol. 2017;28(11):2860‐2865. 29045560 10.1093/annonc/mdx403

[16] Topalian SL , Hodi FS , Brahmer JR , et al. Safety, activity, and immune correlates of anti‐PD‐1 antibody in cancer. N Engl J Med. 2012;366(26):2443‐2454. 22658127 10.1056/NEJMoa1200690 PMC3544539

[17] Hamid O , Robert C , Daud A , et al. Safety and tumor responses with lambrolizumab (anti‐PD‐1) in melanoma. N Engl J Med. 2013;369(2):134‐144. 23724846 10.1056/NEJMoa1305133 PMC4126516

[18] Weber JS , Hodi FS , Wolchok JD , et al. Safety profile of nivolumab monotherapy: a pooled analysis of patients with advanced melanoma. J Clin Oncol. 2017;35(7):785‐792. 28068177 10.1200/JCO.2015.66.1389

[19] Gibson PR , Vaizey C , Black CM , et al. Relationship between disease severity and quality of life and assessment of health care utilization and cost for ulcerative colitis in Australia: a cross‐sectional, observational study. J Crohns Colitis. 2014;8(7):598‐606. 24345767 10.1016/j.crohns.2013.11.017

[20] Friedman CF , Proverbs‐Singh TA , Postow MA . Treatment of the immune‐related adverse effects of immune checkpoint inhibitors: a review. JAMA Oncol. 2016;2(10):1346‐1353. 27367787 10.1001/jamaoncol.2016.1051

[21] Johnson DB , Balko JM , Compton ML , et al. Fulminant myocarditis with combination immune checkpoint blockade. N Engl J Med. 2016;375(18):1749‐1755. 27806233 10.1056/NEJMoa1609214 PMC5247797

[22] van Elsas A , Hurwitz AA , Allison JP . Combination immunotherapy of B16 melanoma using anti‐cytotoxic T lymphocyte‐associated antigen 4 (CTLA‐4) and granulocyte/macrophage colony‐stimulating factor (GM‐CSF)‐producing vaccines induces rejection of subcutaneous and metastatic tumors accompanied by autoimmune depigmentation. J Exp Med. 1999;190(3):355‐366. 10430624 10.1084/jem.190.3.355 PMC2195583

[23] Makarious D , Horwood K , Coward J . Myasthenia gravis: an emerging toxicity of immune checkpoint inhibitors. Eur J Cancer. 2017;82:128‐136. 28666240 10.1016/j.ejca.2017.05.041

[24] Fellner A , Makranz C , Lotem M , et al. Neurologic complications of immune checkpoint inhibitors. J Neurooncol. 2018;137(3):601‐609. 29332184 10.1007/s11060-018-2752-5

[25] Rotz SJ , Leino D , Szabo S , Mangino JL , Turpin BK , Pressey JG . Severe cytokine release syndrome in a patient receiving PD‐1‐directed therapy. Pediatr Blood Cancer. 2017;64(12):e26642. 10.1002/pbc.26642 28544595

[26] Sokal A , de Chou CS , Delyon J , et al. Enteritis without colitis in patients treated with immune checkpoint inhibitors: a tricky diagnosis. Melanoma Res. 2018;28(5):483‐484. 30148767 10.1097/CMR.0000000000000484

[27] Bello E , Cohen JV , Mino‐Kenudson M , Dougan M . Antitumor response to microscopic melanoma in the gastric mucosa mimicking ipilimumab‐induced gastritis. J Immunother Cancer. 2019;7(1):41. 30744698 10.1186/s40425-019-0524-1 PMC6371540

[28] Bergqvist V , Hertervig E , Gedeon P , et al. Vedolizumab treatment for immune checkpoint inhibitor‐induced enterocolitis. Cancer Immunol Immunother. 2017;66(5):581‐592. 28204866 10.1007/s00262-017-1962-6 PMC5406433

[29] Weber J , Thompson JA , Hamid O , et al. A randomized, double‐blind, placebo‐controlled, phase II study comparing the tolerability and efficacy of ipilimumab administered with or without prophylactic budesonide in patients with unresectable stage III or IV melanoma. Clin Cancer Res. 2009;15(17):5591‐5598. 19671877 10.1158/1078-0432.CCR-09-1024

[30] Teulings H‐E , Limpens J , Jansen SN , et al. Vitiligo‐like depigmentation in patients with stage III‐IV melanoma receiving immunotherapy and its association with survival: a systematic review and meta‐analysis. J Clin Oncol. 2015;33(7):773‐781. 25605840 10.1200/JCO.2014.57.4756

[31] Dick J , Lang N , Slynko A , et al. Use of LDH and autoimmune side effects to predict response to ipilimumab treatment. Immunotherapy. 2016;8(9):1033‐1044. 27485076 10.2217/imt-2016-0083

[32] de Malet A , Antoni G , Collins M , et al. Evolution and recurrence of gastrointestinal immune‐related events induced by immune checkpoint inhibitors. Eur J Cancer. 2018;106:106‐114. 30476730 10.1016/j.ejca.2018.10.006

[33] Hopkins AM , Rowland A , Kichenadasse G , et al. Predicting response and toxicity to immune checkpoint inhibitors using routinely available blood and clinical markers. Br J Cancer. 2017;117(7):913‐920. 28950287 10.1038/bjc.2017.274 PMC5625676

[34] Abu‐Sbeih H , Ali FS , Luo W , Qiao W , Raju GS , Wang Y . Importance of endoscopic and histological evaluation in the management of immune checkpoint inhibitor‐induced colitis. J Immunother Cancer. 2018;6(1):95. 30253811 10.1186/s40425-018-0411-1 PMC6156850

[35] Wang Y , Abu‐Sbeih H , Mao E , et al. Endoscopic and histologic features of immune checkpoint inhibitor‐related colitis. Inflamm Bowel Dis. 2018;24(8):1695‐1705. 29718308 10.1093/ibd/izy104

[36] Jain A , Lipson EJ , Sharfman WH , Brant SR , Lazarev MG . Colonic ulcerations may predict steroid‐refractory course in patients with ipilimumab‐mediated enterocolitis. World J Gastroenterol. 2017;23(11):2023‐2028. 28373768 10.3748/wjg.v23.i11.2023 PMC5360643

[37] Brooks GA , Abrams TA , Meyerhardt JA , et al. Identification of potentially avoidable hospitalizations in patients with GI cancer. J Clin Oncol. 2014;32(6):496‐503. 24419123 10.1200/JCO.2013.52.4330 PMC3918534

[38] Lennes I , Eusebio J , Bohlen N , Ruddy M , Ryan DP . Characterization of unplanned 30‐day medical oncology readmissions after discharge at an academic medical center with a comprehensive cancer center. J Clin Oncol. 2016;34(7_suppl):269–269.

[39] Wolchok JD , Chiarion‐Sileni V , Gonzalez R , et al. Overall survival with combined nivolumab and ipilimumab in advanced melanoma. N Engl J Med. 2017;377(14):1345‐1356. 28889792 10.1056/NEJMoa1709684 PMC5706778

[40] Tarhini AA , Zahoor H , Lin Y , et al. Baseline circulating IL‐17 predicts toxicity while TGF‐β1 and IL‐10 are prognostic of relapse in ipilimumab neoadjuvant therapy of melanoma. J Immunother Cancer. 2015;3:39. 26380086 10.1186/s40425-015-0081-1 PMC4570556

[41] Khoja L , Atenafu EG , Templeton A , et al. The full blood count as a biomarker of outcome and toxicity in ipilimumab‐treated cutaneous metastatic melanoma. Cancer Med. 2016;5(10):2792‐2799. 27683208 10.1002/cam4.878 PMC5083732

[42] Eggermont A , Chiarion‐Sileni V , Grob J‐J , et al. Prolonged survival in stage III melanoma with ipilimumab adjuvant therapy. N Engl J Med. 2016;375(19):1845‐1855. 27717298 10.1056/NEJMoa1611299 PMC5648545

[43] Horvat TZ , Adel NG , Dang T‐O , et al. Immune‐related adverse events, need for systemic immunosuppression, and effects on survival and time to treatment failure in patients with melanoma treated with ipilimumab at memorial sloan kettering cancer center. J Clin Oncol. 2015;33(28):3193‐3198. 26282644 10.1200/JCO.2015.60.8448 PMC5087335

[44] Schlager SI , Ohanian SH , Borsos T . Inhibition of antibody‐complement‐mediated killing of tumor cells by hormones. Cancer Res. 1976;36(10):3672‐3677. 182362

[45] Braunschweiger PG , Kumar N , Constantinidis I , et al. Potentiation of interleukin 1 alpha mediated antitumor effects by ketoconazole. Cancer Res. 1990;50(15):4709‐4717. 2369744

[46] Giles AJ , Hutchinson M‐K , Sonnemann HM , et al. Dexamethasone‐induced immunosuppression: mechanisms and implications for immunotherapy. J Immunother Cancer. 2018;6(1):51. 29891009 10.1186/s40425-018-0371-5 PMC5996496

[47] Draghi A , Borch TH , Radic HD , et al. Differential effects of corticosteroids and anti‐TNF on tumor‐specific immune responses: implications for the management of irAEs. Int J Cancer. 2018. 10.1002/ijc.32080 30575963

[48] Arbour KC , Mezquita L , Long N , et al. Impact of baseline steroids on efficacy of programmed cell death‐1 and programmed death‐ligand 1 blockade in patients with non–small‐cell lung cancer. J Clin Oncol. 2018;36(28):2872‐2878. 30125216 10.1200/JCO.2018.79.0006

[49] Faje AT , Lawrence D , Flaherty K , et al. High‐dose glucocorticoids for the treatment of ipilimumab‐induced hypophysitis is associated with reduced survival in patients with melanoma. Cancer. 2018;124(18):3706‐3714. 29975414 10.1002/cncr.31629

[50] National Comprehensive Cancer Network . Clinical Practice Guidelines in Oncology: Cutaneous Melanoma (Version 1.2019). https://www.nccn.org/professionals/physician_gls/pdf/cutaneous_melanoma.pdf Accessed December 7, 2018.

[51] National Comprehensive Cancer Network . Clinical Practice Guidelines in Oncology: Uveal Melanoma (version 1.2018). https://www.nccn.org/professionals/physician_gls/pdf/uveal.pdf Accessed December 7, 2018.

