# Supplementary material for: Colitis after checkpoint blockade: A retrospective cohort study of melanoma patients requiring admission for symptom control
Source: Cancer Med. 2019 Jul 9;8(11):4986–99. doi: 10.1002/cam4.2397 (PMC6718531; doi:10.1002/cam4.2397)
Supplement: Supplementary file 1 [file CAM4-8-4986-s001.pdf]

(a) 8 weeks from initial CPI to earliest corticosteroid at guideline dose: Progression-Free Survival

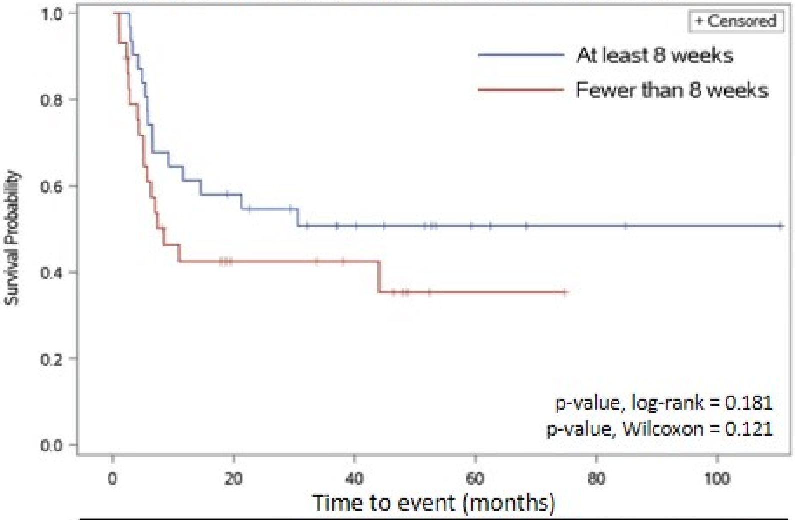

	0 months	20 months	40 months	60 months
No. at risk	60	25	16	5
Fewer than 8 weeks post-CPI	29	8	6	1
At least 8 weeks post-CPI	31	17	10	4

(b) 8 weeks from initial CPI to earliest corticosteroid at guideline dose: Overall Survival

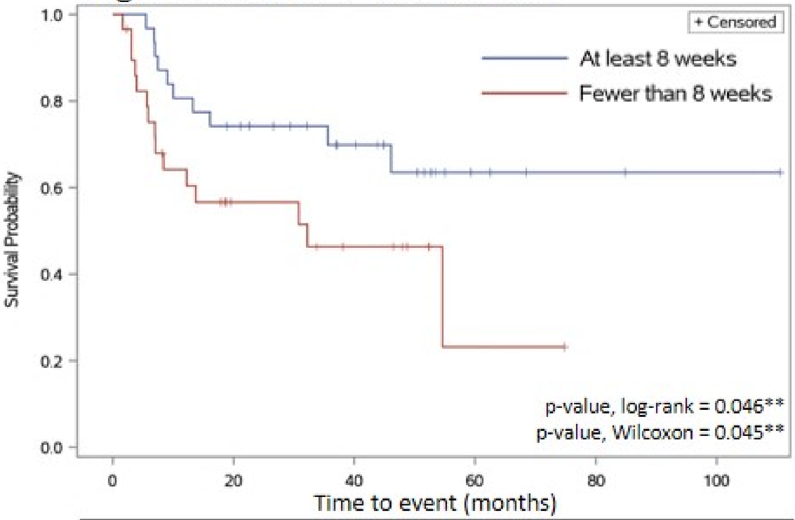

	0 months	20 months	40 months	60 months
No. at risk	60	33	21	5
< 55 days post-CPI	29	11	7	1
At least 55 days post-CPI	31	22	14	4

(c) 64 days from initial CPI to earliest corticosteroid at guideline dose: Progression-Free Survival

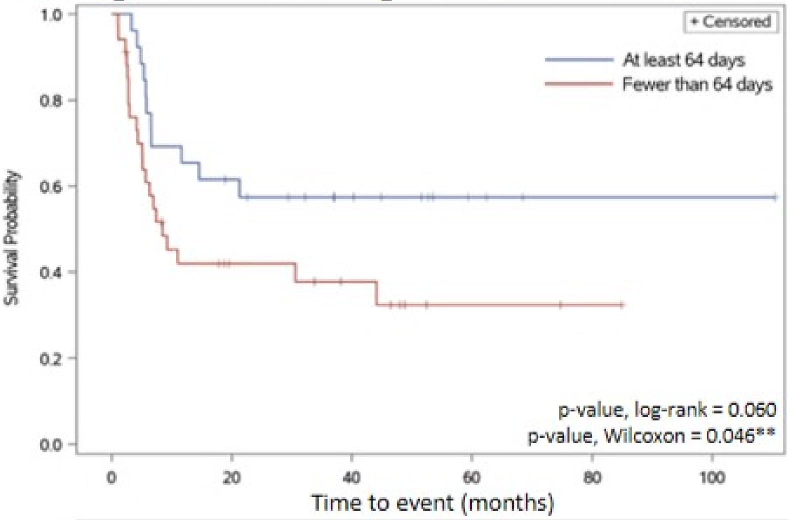

	0 months	20 months	40 months	60 months
No. at risk	60	25	16	5
Fewer than 64 days post-CPI	34	10	7	2
At least 64 days post-CPI	26	15	9	3

(d) 64 days from initial CPI to earliest corticosteroid at guideline dose: Overall Survival

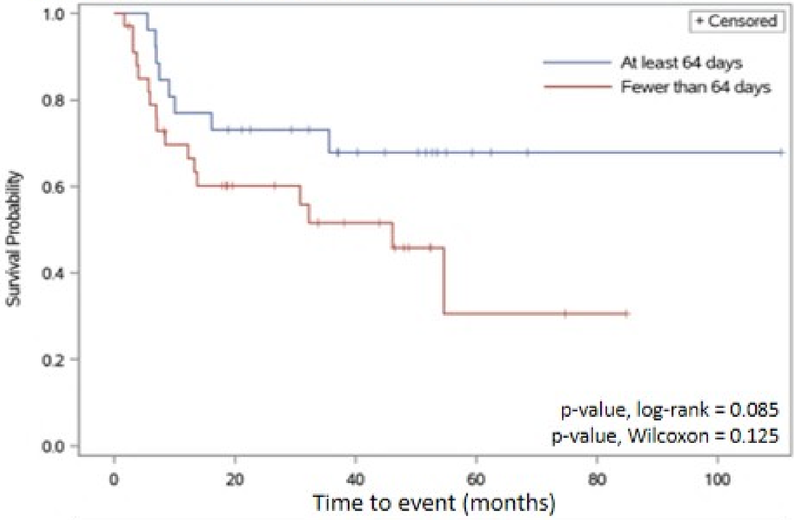

	0 months	20 months	40 months	60 months
No. at risk	60	33	21	5
Fewer than 64 days post-CPI	34	15	10	2
At least 64 days post-CPI	26	18	11	3
