# Supplementary figures and images for: Colitis after checkpoint blockade: A retrospective cohort study of melanoma patients requiring admission for symptom control
Source: Cancer Med. 2019 Jul 9;8(11):4986–99. doi: 10.1002/cam4.2397 (PMC6718531; doi:10.1002/cam4.2397)

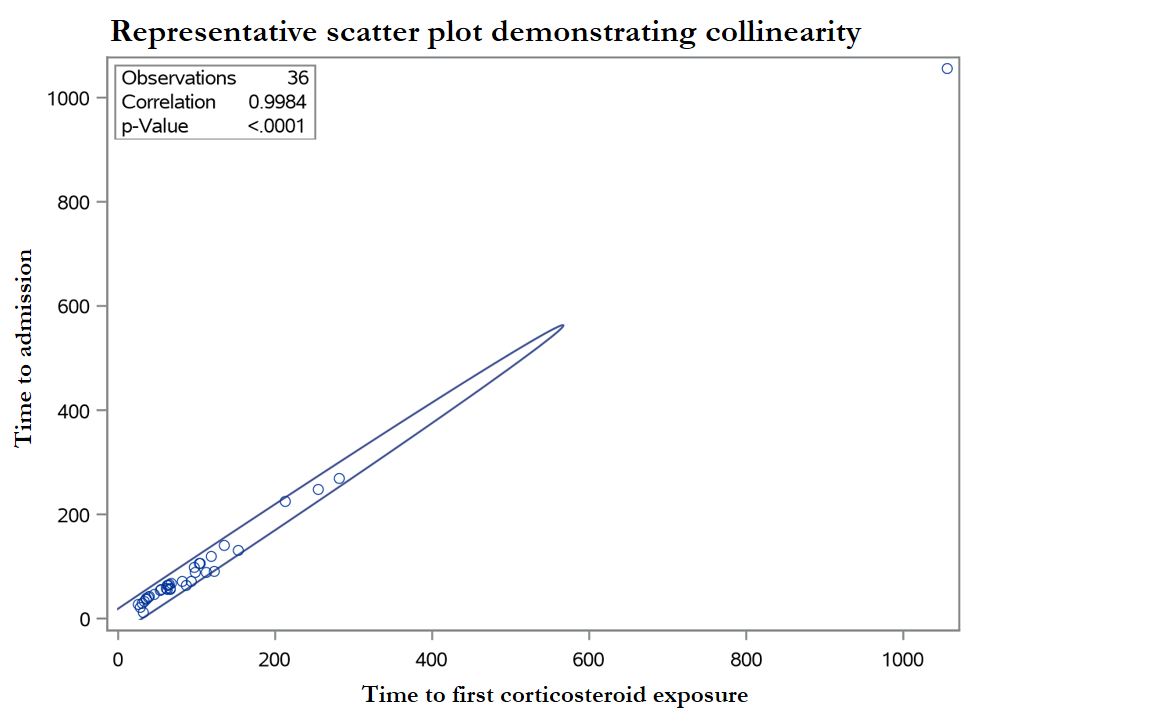

Supplement: Supplementary file 2 [file CAM4-8-4986-s002.tiff]
