# Supplementary material for: Colitis after checkpoint blockade: A retrospective cohort study of melanoma patients requiring admission for symptom control
Source: Cancer Med. 2019 Jul 9;8(11):4986–99. doi: 10.1002/cam4.2397 (PMC6718531; doi:10.1002/cam4.2397)
Supplement: Supplementary file 3 [file CAM4-8-4986-s003.docx]

**Table S1**

Full listing of data collection variables determined *a priori* from clinical experience, sorted alphabetically.

| **Table S1: Full List of Variables Specified prior to Data Collection** |
| --- |
| Age of patient at time of admission |
| Age of patient at time of initial ICI administration |
| Antibiotics within one month of admission |
| Antibiotics within three months of admission |
| CTCAE grade at admission |
| Date of admission |
| Date of birth |
| Date of condition worsening while inpatient, if any |
| Date of discharge |
| Date of infliximab administration |
| Date of last known MGH healthcare contact |
| Date of symptom onset |
| Deceased vs. alive |
| Destination of discharge |
| DFJV Disease Group |
| Dose of alternative second-line immunosuppressive agent |
| Dose of corticosteroids patient was taking upon admission |
| Duration of alternative second-line immunosuppressive agent |
| Duration of inpatient methylprednisolone regimen |
| Duration of prednisolone regimen started inpatient |
| ECOG performance status: at admission |
| ECOG performance status: at initial ICI administration |
| Endoscopy, lower: findings |
| Endoscopy, lower: locations examined |
| Endoscopy, upper: findings |
| Endoscopy, upper: locations examined |
| Fecal studies: calprotectin |
| Fecal studies: fecal leukocyte count |
| Fecal studies: osmolar gap |
| Fecal studies: stool culture |
| Further oncologic outcomes, if rechallenged with ICI |
| Hepatitis B serology screening |
| Histopathologic results from lower endoscopy |
| Histopathologic results from upper endoscopy |
| HLA typing, if applicable |
| ICI rechallenge status |
| ICI regimen |
| CPI-related GEC outcomes, 1 month post-discharge: CT findings |
| CPI-related GEC outcomes, 1 month post-discharge: endoscopic findings |
| CPI-related GEC outcomes, 1 month post-discharge: subjective findings |
| CPI-related GEC outcomes, 12 months post-discharge: CT findings |
| CPI-related GEC outcomes, 12 months post-discharge: endoscopic findings |
| CPI-related GEC outcomes, 12 months post-discharge; subjective findings |
| CPI-related GEC outcomes, 18 months post-discharge: CT findings |
| CPI-related GEC outcomes, 18 months post-discharge: endoscopic findings |
| CPI-related GEC outcomes, 18 months post-discharge: subjective findings |
| CPI-related GEC outcomes, 24 months post-discharge: CT findings |
| CPI-related GEC outcomes, 24 months post-discharge: endoscopic findings |
| CPI-related GEC outcomes, 24 months post-discharge: subjective findings |
| CPI-related GEC outcomes, 3 months post-discharge: CT findings |
| CPI-related GEC outcomes, 3 months post-discharge: endoscopic findings |
| CPI-related GEC outcomes, 3 months post-discharge: subjective findings |
| CPI-related GEC outcomes, 6 months post-discharge: CT findings |
| CPI-related GEC outcomes, 6 months post-discharge: endoscopic findings |
| CPI-related GEC outcomes, 6 months post-discharge: subjective findings |
| CPI-related GEC outcomes, 9 months post-discharge: CT findings |
| CPI-related GEC outcomes, 9 months post-discharge: endoscopic findings |
| CPI-related GEC outcomes, 9 months post-discharge: subjective findings |
| Infectious disease studies: Adenovirus |
| Infectious disease studies: Clostridium difficile assay |
| Infectious disease studies: Cryptosporidium |
| Infectious disease studies: Cytomegalovirus |
| Infectious disease studies: Giardia |
| Infectious disease studies: Helicobacter pylori stool antigen/breath test |
| Infectious disease studies: Microsporidium |
| Infectious disease studies: other testing |
| Infectious disease studies: Rotavirus |
| Infectious disease studies: stool ova and parasites examination |
| Initial ICI exposure |
| Laboratory testing: Albumin (upon admission and additionally if required critical care) |
| Laboratory testing: Blood urea nitrogen (upon admission and additionally if required critical care) |
| Laboratory testing: C-reactive protein (upon admission and additionally if required critical care) |
| Laboratory testing: Erythrocyte sedimentation rate (upon admission and additionally if required critical care) |
| Laboratory testing: Hematocrit (upon admission and additionally if required critical care) |
| Laboratory testing: Hemoglobin (upon admission and additionally if required critical care) |
| Laboratory testing: Lactate (upon admission and additionally if required critical care) |
| Laboratory testing: Lactate dehydrogenase (upon admission and additionally if required critical care) |
| Laboratory testing: Leukocyte count (upon admission and additionally if required critical care) |
| Laboratory testing: Relative lymphocyte count (upon admission and additionally if required critical care) |
| Laboratory testing: Serum chloride (upon admission and additionally if required critical care) |
| Laboratory testing: Serum creatinine (upon admission and additionally if required critical care) |
| Laboratory testing: Serum ferritin (upon admission and additionally if required critical care) |
| Laboratory testing: Serum iron (upon admission and additionally if required critical care) |
| Laboratory testing: Serum platelets (upon admission and additionally if required critical care) |
| Laboratory testing: Serum potassium (upon admission and additionally if required critical care) |
| Laboratory testing: Serum sodium (upon admission and additionally if required critical care) |
| Laboratory testing: Tissue transglutaminase IgA (upon admission and additionally if required critical care) |
| Laboratory testing: Total protein (upon admission and additionally if required critical care) |
| Laboratory testing: Vitamin B12 |
| Length of stay |
| Lower endoscopy modality |
| Maximum dose of inpatient methylprednisolone |
| Maximum dose of inpatient prednisolone |
| Maximum dose of TNFαi |
| Maximum irAE severity grade |
| Medical Record Number |
| Most intensive treatment location: general ward vs intensive care |
| New GEC recurrence, if rechallenged with ICI |
| New irAE occurrence, if rechallenged with ICI |
| New irAE severity, if rechallenged with ICI |
| Number of prior therapies |
| Oncologic outcomes at 18 months post-discharge, 18mo |
| Oncologic outcomes at 24 months post-discharge |
| Oncologic outcomes at nine months post-discharge |
| Oncologic outcomes at one month post-discharge |
| Oncologic outcomes at one year post-discharge |
| Oncologic outcomes at six months post-discharge |
| Oncologic outcomes at three months post-discharge |
| Outcome of new irAE, if rechallenged with ICI |
| Pneumocystis jirovecii pneumonia prophylaxis |
| Presence and location of gastrointestinal metastases at time of admission |
| Prior oncologic regimens |
| Purified protein derivative testing/other tuberculosis screening test result |
| Putative location of inflammation at admission |
| Radiographic imaging: bowel wall thickening |
| Radiographic imaging: diffuse inflammation |
| Radiographic imaging: fluid-filled distention |
| Radiographic imaging: mesenteric vessel engorgement |
| Radiographic imaging: perf (worst) |
| Radiographic imaging: segmental inflammation a/w diverticulosis |
| Redosing of TNFαi |
| Requirement for critical care at admission |
| Sex |
| Significant tumor cytogenetic abnormalities |
| Status of oncologic response at time of discharge |
| Study identifier |
| Symptoms and signs that prompted application of critical care |
| Symptoms/signs at presentation |
| Time from initial ICI administration to admission |
| Time from initial ICI administration to hepatitis B serology testing |
| Time from initial ICI administration to symptom onset |
| Time from initial ICI administration to tuberculosis screening |
| Time from symptom onset to admission |
| Time to corticosteroid administration or other GEC-related intervention from admission |
| Time to earliest methylprednisolone dose from admission |
| Time to ICI rechallenge from last ICI dose prior to most recent admission |
| Treatment regimen for new irAE, if rechallenged with ICI |
| Tuberculosis screening |
| Tumor stage |
| Type of irAE |
| Upper endoscopy modality |
| Use of alternative second-line immunosuppression, and if so agent used |
| Use of corticosteroids upon admission |
| Use of inpatient antacid regimen |
| Use of inpatient ciprofloxacin or metronidazole |
| Use of methylprednisolone prior to TNFαi |
| Use of oral corticosteroids prior to methylprednisolone |
| Use of oral corticosteroids prior to TNFαi |
| Use of other immunosuppression prior to alternative agent |
| Use of pre-ICI antacid |
| Weight at initial ICI administration |
| Weight change |
| Weight upon admission |
