# Supplementary material for: Colitis after checkpoint blockade: A retrospective cohort study of melanoma patients requiring admission for symptom control
Source: Cancer Med. 2019 Jul 9;8(11):4986–99. doi: 10.1002/cam4.2397 (PMC6718531; doi:10.1002/cam4.2397)
Supplement: Supplementary file 4 [file CAM4-8-4986-s004.docx]

**Table S2**

Additional presentation features of CPI-related GEC and results from diagnostic testing, including full routine chemistries and full complete blood count.

| **Table S2: Additional features of CPI-related GEC presentation and initial diagnostic approach** | | | | | | |
| --- | --- | --- | --- | --- | --- | --- |
|  | | Overall | No use of second-line immunosuppression | Use of second-line immunosuppression | p-value | |
| Laboratory results at admission: mean +/- SD | | | | | | |
|  | Serum sodium (mmol/L) | 135 +/- 4 | 136.1 +/- 2.7 | 134.9 +/- 4.4 | | 0.133 |
|  | Serum potassium (mmol/L) | 3.9 +/- 0.6 | 4.0 +/- 0.6 | 3.8 +/- 0.6 | | 0.273 |
|  | Serum chloride (mmol/L) | 99 +/- 5 | 100 +/- 3 | 99 +/- 5 | | 0.354 |
|  | Blood urea nitrogen (mg/dL) | 21.7 +/- 15.1 | 21.4 +/- 16.2 | 22.0 +/- 14.4 | | 0.850 |
|  | Serum creatinine (mg/dL) | 1.2 +/- 1.0 | 1.2 +/- 1.2 | 1.3 +/- 1.0 | | 0.717 |
|  | Lactate (mmol/L) | 1.7 +/- 1.1 | 1.8 +/- 1.0 | 1.6 +/- 1.1 | | 0.766 |
|  | Leukocytes (K cells/mL) | 9.04 +/- 3.81 | 8.95 +/- 4.37 | 9.11 +/- 3.42 | | 0.850 |
|  | Hgb (g/dL) | 12.8 +/- 2.0 | 12.5 +/- 1.6 | 13.0 +/- 2.2 | | 0.247 |
|  | Hct (%) | 38.3 +/- 5.1 | 37.6 +/- 4.1 | 38.7 +/- 5.7 | | 0.300 |
|  | Plt (K cells/mL) | 273 +/- 90 | 263 +/- 87 | 280 +/- 92 | | 0.389 |
| Diagnostic studies on admission | | | | | | |
|  | EGD signs of GI inflammation | 8/12 (66.7%) | 4/4 (100.0%) | 4/8 (50.0%) | | 0.208 |
|  | Lower endoscopy signs of GI inflammation | 49/67 (73.1%) | 20/29 (69.0%) | 29/38 (76.3%) | | 0.501 |
| The p-value was calculated by ANOVA for numerical covariates and chi-square test or Fisher’s exact for categorical covariates, where appropriate.  SD: standard deviation  IQR: interquartile range  ECOG: Eastern cooperative oncology group  CTCAE: common terminology criteria for adverse events  LDH: lactate dehydrogenase  GI: gastrointestinal  EGD: esophagogastroduodenoscopic  **Statistically significant at α<0.05 | | | | | | |
